# Global burden and risk factors of childhood cardiovascular disease (1990–2021)

**DOI:** 10.3389/fpubh.2025.1543044

**Published:** 2025-07-03

**Authors:** Caixia Hou, Nan Zhang, Chao Liu, Weijun Gao

**Affiliations:** ^1^Qingdao Central Hospital, University of Health and Rehabilitation Sciences, Qingdao, China; ^2^Faculty of Environmental Engineering, The University of Kitakyushu, Kitakyushu, Japan; ^3^Innovation Institute for Sustainable Maritime Architecture Research and Technology, Qingdao University of Technology, Qingdao, China

**Keywords:** childhood cardiovascular disease, health burden, EAPC, health inequality, GBD

## Abstract

**Background:**

Childhood cardiovascular disease (CCVD) is a significant global health threat, with early risk factor accumulation potentially exerting profound impacts on cardiovascular health in adulthood. However, global data analyses on the burden of CCVD remain limited, particularly regarding variations across socio-demographic index (SDI) levels and effects of major risk factors.

**Objective:**

This study aimed to investigate worldwide patterns in the incidence and mortality of CCVD between 1990 and 2021 and examine how these trends vary by SDI, gender, age, and major environmental risk factors.

**Methods:**

This study analyzed the incidence, mortality, and estimated annual percentage change (EAPC) of CCVD in children aged 0 to 14 years during 1990–2021. Descriptive statistics, group comparisons, and correlation analyses were employed to evaluate the impact of SDI, gender, age, and environmental risks on the disease burden.

**Results:**

The global CCVD incidence rose from 1,486,136.37 cases (95% UI: 1,115,077.02–1,959,529.28) in 1990 to 1,861,693.20 cases (95% UI: 1,335,751.17–2,531,859.51) in 2021, an increase of approximately 25.27%, with an EAPC of 0.43 (95% UI: 0.35–0.51). Incidence rates increased continuously in low and low-middle SDI regions, whereas they showed a marked decline in regions with high and high-middle SDI levels. CCVD mortality decreased markedly in high-SDI regions but remained persistently high in low SDI regions. Gender and age analyses revealed higher incidence and mortality rates among female children compared to males, with the 10–14 age group being the most affected. Low temperatures were identified as a primary driver of CCVD burden, particularly in low-SDI regions.

**Conclusion:**

The global CCVD burden exhibits significant inequalities, largely driven by disparities in public health resource levels across regions with varying SDI. These findings highlight persistent global health inequalities and underscore the need for region-specific interventions, especially in low-SDI regions where the CCVD burden is rising.

## 1 Introduction

Childhood cardiovascular disease (CCVD) is increasingly recognized as a pressing issue in global health discourse (1). While the likelihood of children experiencing conditions such as myocardial infarction or stroke is relatively low, the accumulation of risk factors during childhood significantly increases the probability of developing cardiovascular diseases (CVD) in adulthood. Unhealthy lifestyle habits, overnutrition or malnutrition, and hormonal imbalances during childhood are key predictors of future CVD risk (2, 3). Therefore, understanding how the global health burden of CCVD evolves over time and across regions is crucial.

Socioeconomic development levels, reflected by the Sociodemographic Index (SDI), are strongly associated with the global burden of CCVD (4). Nations with elevated SDI scores typically possess stronger health infrastructures and more effective early intervention measures, leading to comparatively reduced rates of CVD incidence and mortality. Conversely, countries with low SDI frequently face a heavier CVD burden as a result of scarce medical resources and increased vulnerability to risk factors (4). A similar pattern was observed by Roth et al. (5) in their study of CVD burdens across U. S. states. Similarly, Sun et al. (6) and Tong et al. (7) highlighted the severe CVD burden among individuals aged 15–39, influenced by age, gender, SDI, and regional or national differences. Despite the increasing recognition of early cardiovascular risk, comprehensive global assessments of CCVD incidence, mortality, and risk patterns across socio-demographic and geographic groups remain scarce.

Using global health data spanning 1990 to 2021, this study conducted a comprehensive analysis of the temporal patterns and geographical variation in CCVD incidence and mortality. It evaluated the impact of key factors such as SDI, environmental risks, and non-optimal temperatures on disease burden. Unlike previous studies that primarily focused on adult populations or specific regions, this study represents the earliest comprehensive evaluation of CCVD on a global scale, employing indicators such as the estimated annual percentage change (EAPC) to quantitatively analyze trends across 204 countries and territories by age, gender, and SDI level. The findings not only address a significant gap in the global assessment of CCVD burden but also uncover the underlying socio-economic and environmental drivers of disease trends. This study offers novel scientific insights and practical guidance for formulating targeted interventions in child health, optimizing healthcare resource distribution, and promoting equity in global public health outcomes.

## 2 Methods

### 2.1 Data collection

This study is a secondary data analysis based on the Global Burden of Disease (GBD) 2021 database, employing a retrospective ecological design to assess CCVD burden across time and regions. This study drew on CVD data from GBD 2021, covering incidence and mortality among children aged 0 to 14 across 204 countries from 1990 to 2021. The study analyzed the current status and predicted trends of CCVD development through global incidence and mortality rates. To better understand the CCVD burden across different childhood stages, children aged 0–14 years were further categorized into four groups: <1 year, 1–4 years, 5–9 years, and 10–14 years (8). As the data were obtained from publicly available databases and did not involve personal information, ethical approval was waived.

### 2.2 SDI

According to indicators like education and income per capita (9), 204 countries are classified into five SDI levels: low, low-middle, middle, high-middle, and high. An SDI score ranges from 0 to 1, where a higher score denotes a higher degree of socioeconomic progress (10).

### 2.3 Statistical analysis

This study comprehensively analyzed the spatiotemporal characteristics of global CCVD incidence and mortality from 1990 to 2021. First, the EAPC served as a metric to evaluate the mean yearly variation in the CCVD burden, highlighting overall trends and regional differences. The calculation of EAPC followed the methodology outlined in Equations 1, 2 (11). Second, the study used regression analysis to examine how SDI correlates with the CCVD burden, revealing the impact of socioeconomic development levels. In addition, the contributions of primary risk factors to CCVD incidence and mortality were analyzed in this study, analyzing their distribution and dynamic changes across regions with different SDI levels. All analyses were performed in R (V.4.2.1) and JD_GBDR (V2.24, Jingding Medical Technology Co., Ltd.). *p* < 0.05 was considered statistically significant.


(1) y=α+βχ+ε

(2) EAPC=100×(exp(β)−1)

Log-linear regression models were employed to compute the EAPC, which quantifies average yearly variations in incidence and mortality rates. An upward trend is reflected by a positive EAPC, whereas a negative value signifies a decline.

## 3 Results

### 3.1 Global and regional patterns and yearly variations in CCVD incidence (1990–2021)

At the global level, the incidence of CCVD consistently increased over time, rising from 1,486,136.37 cases (95% UI: 1,115,077.02–1,959,529.28) in 1990 to 1,861,693.20 cases (95% UI: 1,335,751.17–2,531,859.51) in 2021, representing an increase of approximately 25.27%. The incidence rate grew by 7.09, with an EAPC of 0.43 (95% UI: 0.35–0.51, refer to Table 1). Incidence cases and rates declined in regions classified as having high, high-middle, and middle SDI levels, with EAPCs of −0.77 (95% UI: −0.94 to −0.60), −0.83 (95% UI: −0.90 to −0.76), and −0.22 (95% UI: −0.30 to −0.13), respectively. Conversely, regions with low and low-middle SDI levels showed upward trends, with EAPCs of 0.66 (95% UI: 0.56–0.77) and 0.45 (95% UI: 0.40–0.49), respectively. Eastern and Western Southern Sub-Saharan Africa showed notable increases in incidence rates, whereas East Asia, Western Europe, and the high-income Asia-Pacific regions experienced declining trends. Overall, during 1990–2021, the global incidence of CCVD exhibited a slight increase, with notable differences in trends across various SDI levels and regions.

**Table 1:** Global and regional variations in CCVD incidence (1990–2021).

Region	CCVD incidence / 100,000 (95%UI)					
	1990		2021		1990–2021	
	Incidence cases	Incidence rate	Incidence cases	Incidence rate	Cases change	EAPC^a^
Global	1486136.37 (1115077.02–1959529.28)	85.45 (64.12–112.67)	1861693.20 (1335751.17–2531859.51)	92.54 (66.39–125.85)	25.27 (18.27–30.54)	0.43 (0.35–0.51)
SDI						
High	55653.70 (45547.02–69700.17)	29.95 (24.51–37.51)	45330.88 (36999.73–56659.82)	26.27 (21.44–32.84)	−18.55 (−20.80--16.59)	−0.77 (−0.94--0.60)
High middle	285675.75 (205797.27–385543.72)	124.80 (89.90–168.42)	636597.86 (442425.18–876581.56)	138.32 (96.13–190.47)	−33.96 (−36.51--31.82)	−0.83 (−0.90--0.76)
Middle	570854.17 (425900.90–754006.64)	98.90 (73.79–130.63)	504378.22 (362860.42–681816.41)	88.98 (64.01–120.28)	−11.64 (−15.75--8.84)	−0.22 (−0.30--0.13)
Low middle	170106.22 (139292.33–208137.77)	62.17 (50.91–76.07)	112339.58 (88791.33–141452.90)	48.65 (38.46–61.26)	39.46 (33.53–44.24)	0.66 (0.56–0.77)
Low	402697.26 (292397.76–543353.12)	85.30 (61.93–115.09)	561620.15 (394482.23–773725.05)	96.86 (68.03–133.44)	122.84 (114.23–130.01)	0.45 (0.40–0.49)
Regions						
Central Sub-Saharan Africa	47440.04 (33381.30–66257.88)	187.52 (131.95–261.90)	114437.34 (78009.84–162311.23)	195.01 (132.94–276.60)	141.23 (127.71–153.13)	0.04 (0.01–0.08)
Central Latin America	44386.16 (34124.15–57404.04)	68.94 (53.00–89.16)	42503.14 (31768.21–56232.42)	66.95 (50.04–88.58)	−4.24 (−7.93--0.96)	−0.18 (−0.22--0.14)
East Asia	381845.53 (293719.81–491472.85)	115.77 (89.05–149.01)	234379.03 (171543.69–311139.53)	87.67 (64.16–116.38)	−38.62 (−42.20--35.76)	−0.77 (−0.89--0.64)
South Asia	288746.73 (205606.03–389934.21)	66.63 (47.44–89.98)	372928.59 (253625.61–517464.47)	73.55 (50.02–102.06)	29.15 (22.43–34.91)	1.04 (0.74–1.33)
Eastern Sub-Saharan Africa	141903.57 (98973.46–193835.20)	156.68 (109.28–214.02)	310146.41 (208445.72–432494.39)	173.82 (116.82–242.39)	118.56 (109.32–125.81)	0.42 (0.38–0.45)
Western Sub-Saharan Africa	126090.34 (95637.02–165246.14)	143.48 (108.83–188.04)	309721.18 (226562.54–418223.69)	144.21 (105.49–194.74)	145.63 (135.16–153.83)	−0.05 (−0.07--0.03)
North Africa and Middle East	100523.58 (79271.48–127757.01)	71.55 (56.43–90.94)	129603.07 (96930.06–169609.64)	70.70 (52.87–92.52)	28.93 (21.60–35.76)	−0.12 (−0.19--0.06)
Southeast Asia	104709.38 (82130.14–130872.91)	61.32 (48.10–76.65)	105044.87 (80757.20–135034.52)	60.84 (46.77–78.21)	0.32 (−3.60–3.79)	0.02 (−0.06–0.10)
Central Asia	23213.82 (16735.36–31513.79)	92.89 (66.96–126.10)	26172.18 (18654.52–35791.03)	94.57 (67.40–129.32)	12.74 (8.97–16.17)	−0.13 (−0.28–0.01)
High-income North America	19387.22 (15289.75–24807.47)	31.43 (24.79–40.22)	17255.28 (13809.70–21799.07)	26.30 (21.05–33.22)	−11.00 (−15.96--6.34)	−1.31 (−1.65--0.98)
Andean Latin America	17976.63 (12644.88–24572.55)	121.04 (85.14–165.45)	21839.81 (15102.53–30297.91)	120.70 (83.46–167.44)	21.49 (16.87–26.24)	−0.04 (−0.06--0.02)
Tropical Latin America	76214.40 (52522.92–105972.55)	142.15 (97.96–197.66)	67665.48 (46304.75–93866.54)	134.81 (92.25–187.01)	−11.22 (−13.15--8.92)	−0.24 (−0.27--0.21)
Oceania	3548.59 (2674.05–4636.17)	132.42 (99.78–173.00)	7006.43 (5255.35–9208.76)	137.90 (103.43–181.24)	97.44 (89.80–107.72)	0.13 (0.06–0.20)
Southern Sub-Saharan Africa	35021.72 (23930.65–48797.16)	169.27 (115.67–235.86)	41530.23 (27947.42–58351.13)	172.57 (116.13–242.46)	18.58 (15.09–22.05)	−0.07 (−0.13--0.01)
Western Europe	17006.42 (13401.24–21542.73)	23.95 (18.87–30.33)	14742.29 (11732.86–18358.79)	21.64 (17.22–26.95)	−13.31 (−16.33--10.46)	−0.45 (−0.55--0.36)
Caribbean	13800.10 (10002.98–18610.26)	120.92 (87.65–163.07)	14694.10 (10403.54–20041.49)	127.72 (90.43–174.20)	6.48 (1.66–10.53)	0.12 (0.10–0.15)
Eastern Europe	12641.98 (9940.79–16331.70)	24.57 (19.32–31.74)	8377.72 (6606.65–10689.19)	23.64 (18.64–30.16)	−33.73 (−36.18--31.47)	−0.24 (−0.29--0.19)
Southern Latin America	12278.92 (9057.55–16401.58)	82.26 (60.68–109.88)	12501.01 (8972.19–17027.00)	86.24 (61.90–117.46)	1.81 (−4.04–7.98)	0.15 (0.11–0.19)
High-income Asia Pacific	10507.20 (8313.06–13327.57)	29.85 (23.62–37.86)	6025.49 (4670.46–7771.88)	26.87 (20.83–34.66)	−42.65 (−44.94--40.69)	−0.52 (−0.59--0.44)
Central Europe	8039.08 (6514.05–9928.54)	27.27 (22.09–33.67)	4101.99 (3287.86–5145.30)	23.17 (18.57–29.07)	−48.97 (−50.73--47.21)	−0.70 (−0.77--0.64)
Australasia	854.97 (674.51–1096.19)	18.64 (14.71–23.90)	1017.58 (810.98–1266.35)	17.76 (14.15–22.10)	19.02 (10.40–25.31)	−0.18 (−0.23--0.13)

### 3.2 Gender distribution and trends in CCVD incidence rates (1990–2021)

**Figure 1:**
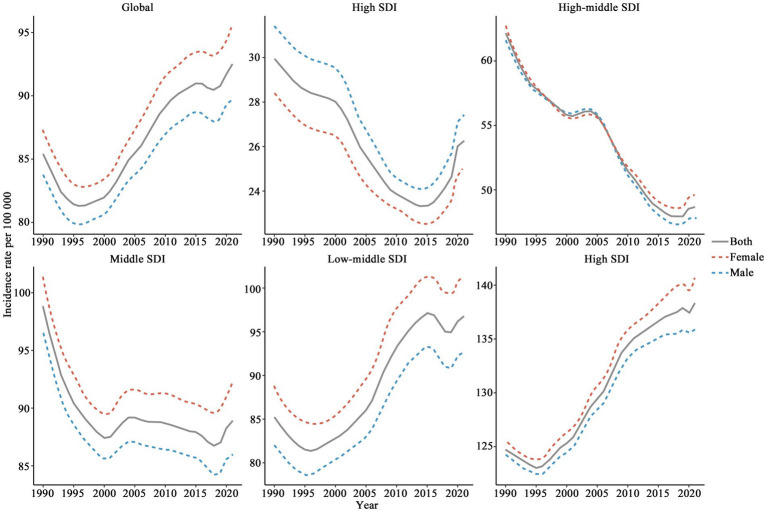
Changes in CCVD incidence rates across global and SDI levels (1990–2021).

The global CCVD incidence rate initially decreased from 1990 to 1996 (see Figure 1), followed by a gradual increase, reaching a higher level in 2021 (92.54, 95% UI: 66.39–125.85). Incidence rates were consistently higher in females than in males, with the gap between genders expanding over the study period. Regions with high SDI maintained comparatively low incidence rates (29.95–23.33, 95% UI: 19.14–37.51) and showed a steady decline since 1990, stabilizing at a low level in 2021 (26.27, 95% UI: 21.44–32.50). The variation in incidence rates between genders in these regions was minimal, with females slightly lower than males. A consistent decline in incidence rates was observed in high-middle SDI regions from 1990 to 2020, with overlapping trends across genders, implying no substantial male–female variation. In Middle SDI regions, the incidence rate among females was consistently greater than that among males, with the gender gap gradually widening. The trend in Low-middle SDI regions closely mirrored the global pattern. However, in low SDI regions, the incidence rate was the highest and exhibited sustained growth from 1990, culminating in a peak in 2021 (138.32, 95% UI: 96.13–190.47). Overall, the global CCVD incidence rate exhibited an upward trend, with females bearing a heavier burden.

### 3.3 Trends in CCVD incidence rates across different age groups (1990–2021)

**Figure 2:**
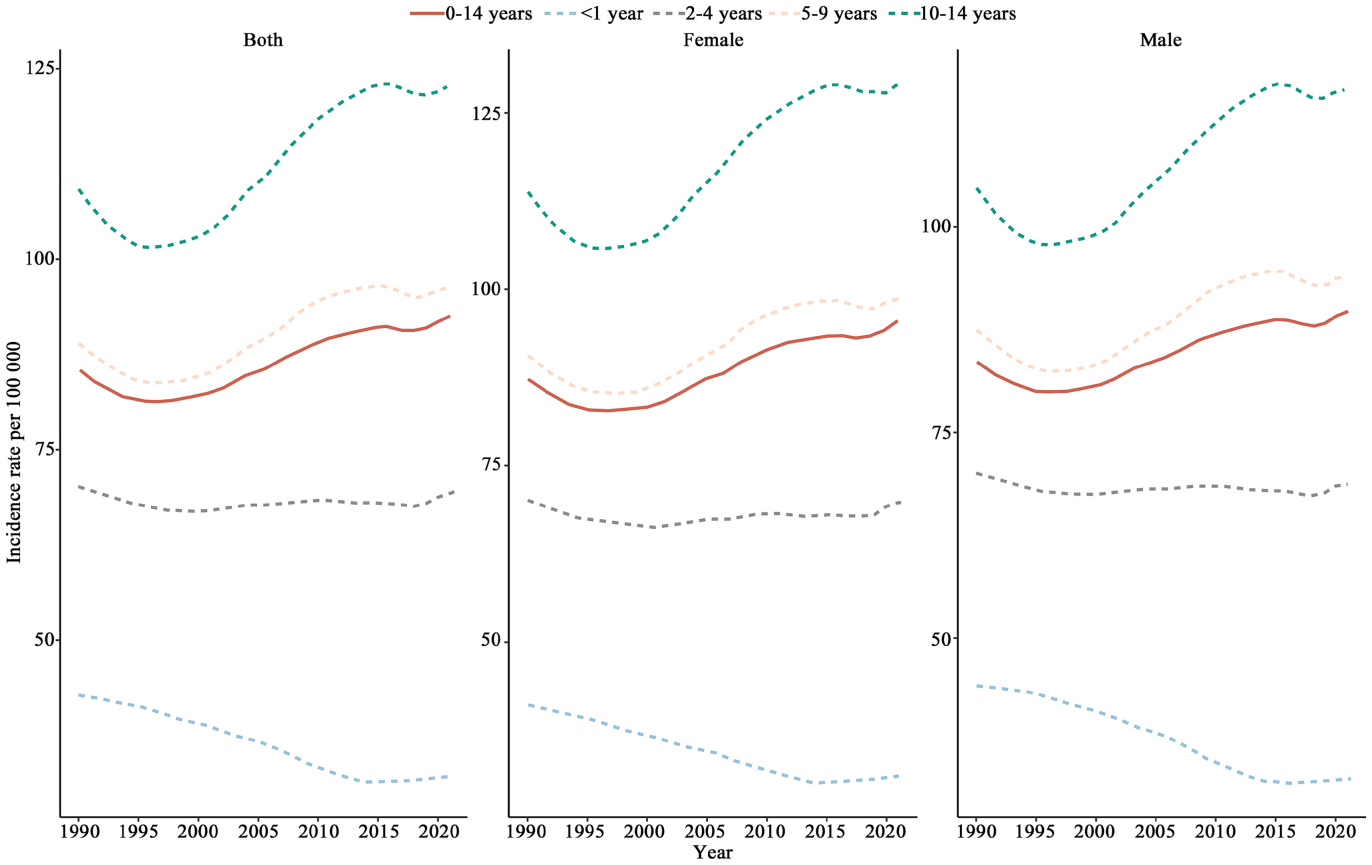
Trends in CCVD incidence rates across different age groups (1990–2021).

The incidence rate of CCVD in children below 1 year of age showed a continuous decline, while the incidence rates for children aged 5–14 exhibited an upward trend after 1996 (see Figure 2). Children aged 10–14 were identified as having the highest CCVD incidence among all age groups. Higher incidence rates were observed among females relative to males across all age groups, with the most pronounced gap occurring in individuals aged 10 to 14.

### 3.4 Geographical distribution of global CCVD incidence rates and EAPC

**Figure 3:**
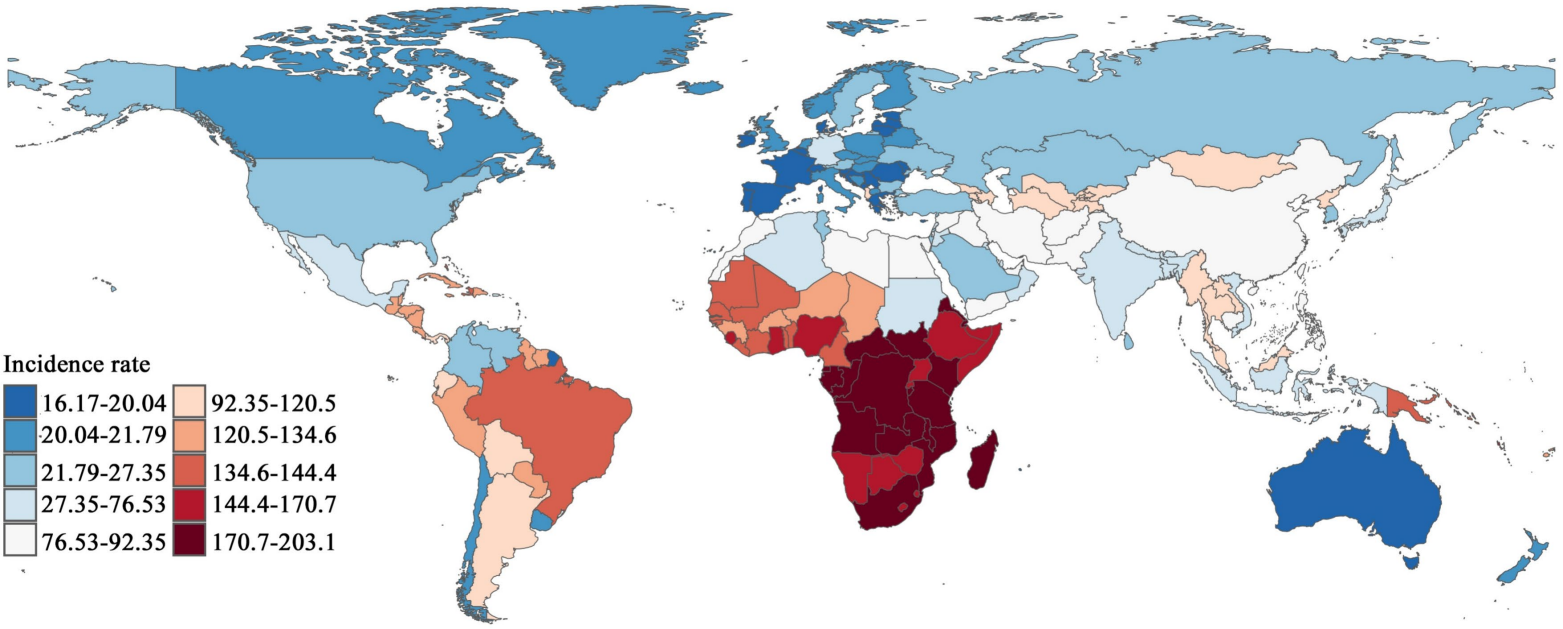
Geographical distribution in global CCVD incidence rates (1990–2021).

In 2021, the geographical distribution of CCVD incidence rates displayed notable variations across regions, as shown in Figure 3. Southern Sub-Saharan Africa featured a significant concentration of dark red areas, Congo reported the highest incidence rate, reaching 203.09 (95% UI: 139.05–289.53). Parts of South Asia and the Middle East also exhibited high incidence rates. In certain areas of South America, particularly in low-income countries, the incidence rate was relatively high, appearing in shades close to orange. Conversely, countries such as Spain and Switzerland reported notably lower incidence rates, at 16.65 (95% UI: 13.03–20.84) and 16.17 (95% UI: 12.43–20.54), respectively. Overall, most countries in Africa, South Asia, and Latin America experienced higher incidence rates, while lower rates were observed in North America, Europe, Australia, and certain East Asian countries.

**Figure 4:**
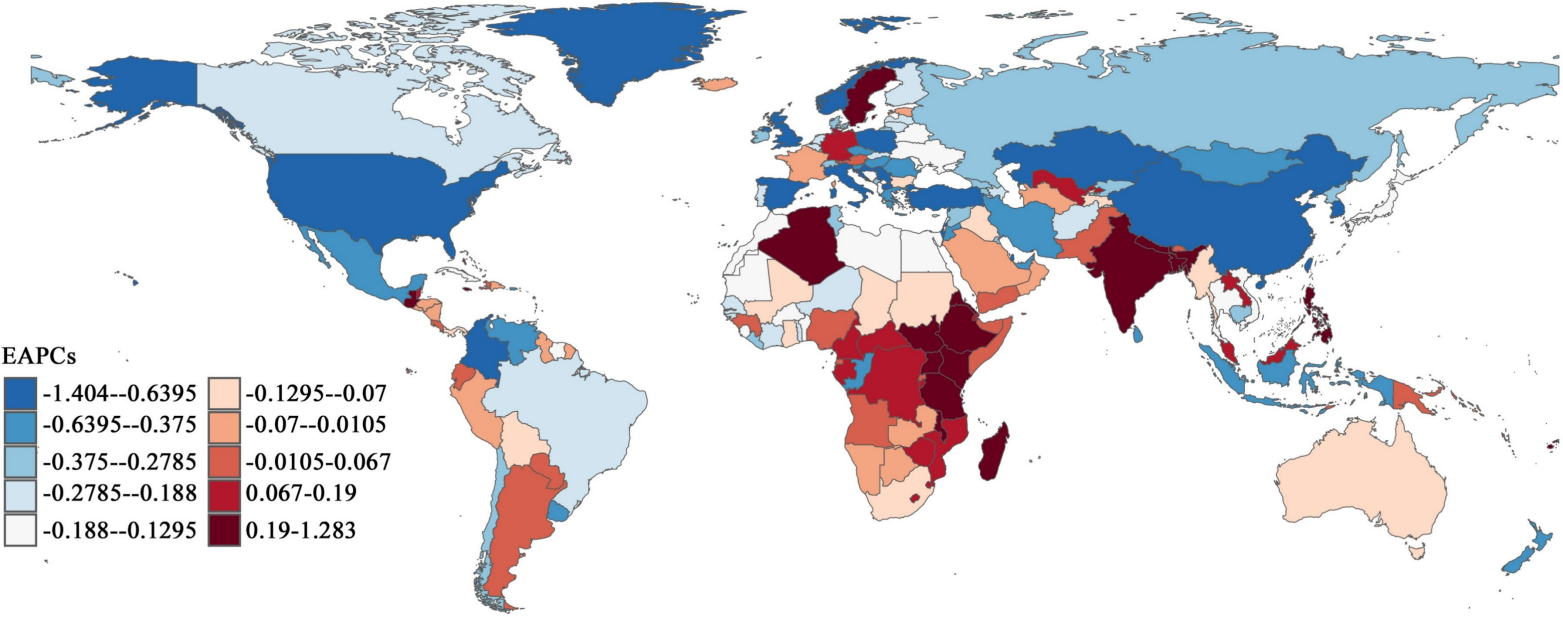
Geographical distribution of EAPC in global CCVD incidence rates (1990–2021).

As shown in Figure 4, Southern Sub-Saharan Africa, South Asia, and certain Middle Eastern regions demonstrated notable upward trends in CCVD incidence EAPC values. The most substantial increases and highest concentrations of dark red zones were found in Central Africa and South Asia. India recorded the highest EAPC in incidence rate (1.29), followed by Fiji (1.76). Among the 204 countries analyzed, 69.61% showed an EAPC in incidence rates below zero, mainly distributed across North America, Europe, and East Asia. North America and Western Europe had the largest declines, with the highest concentration of dark blue areas. Overall, developed countries continued to experience declines in CCVD incidence rates, while an upward trend was observed in low- and lower-middle-income countries, reflecting the persistent global public health inequities. Notably, some countries with relatively low incidence rates showed worsening trends. For example, Sweden reported an incidence rate of 25.06 (95% UI: 19.11–33.14) but exhibited a positive EAPC of 0.39.

### 3.5 Association analysis of CCVD incidence rate, SDI, and EAPC

**Figure 5:**
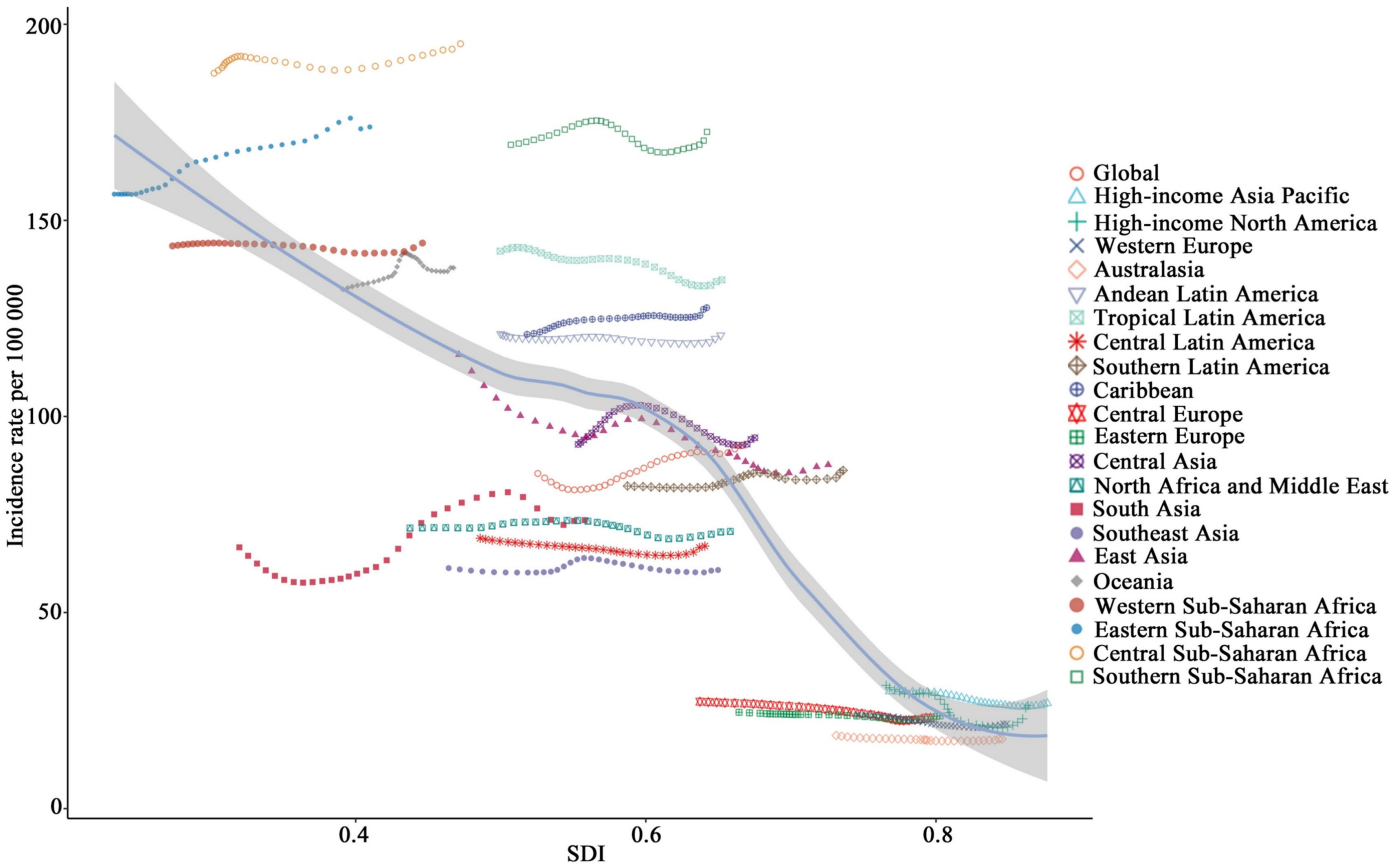
The association between incidence rates and SDI across 21 regions (1990–2021).

**Figure 6:**
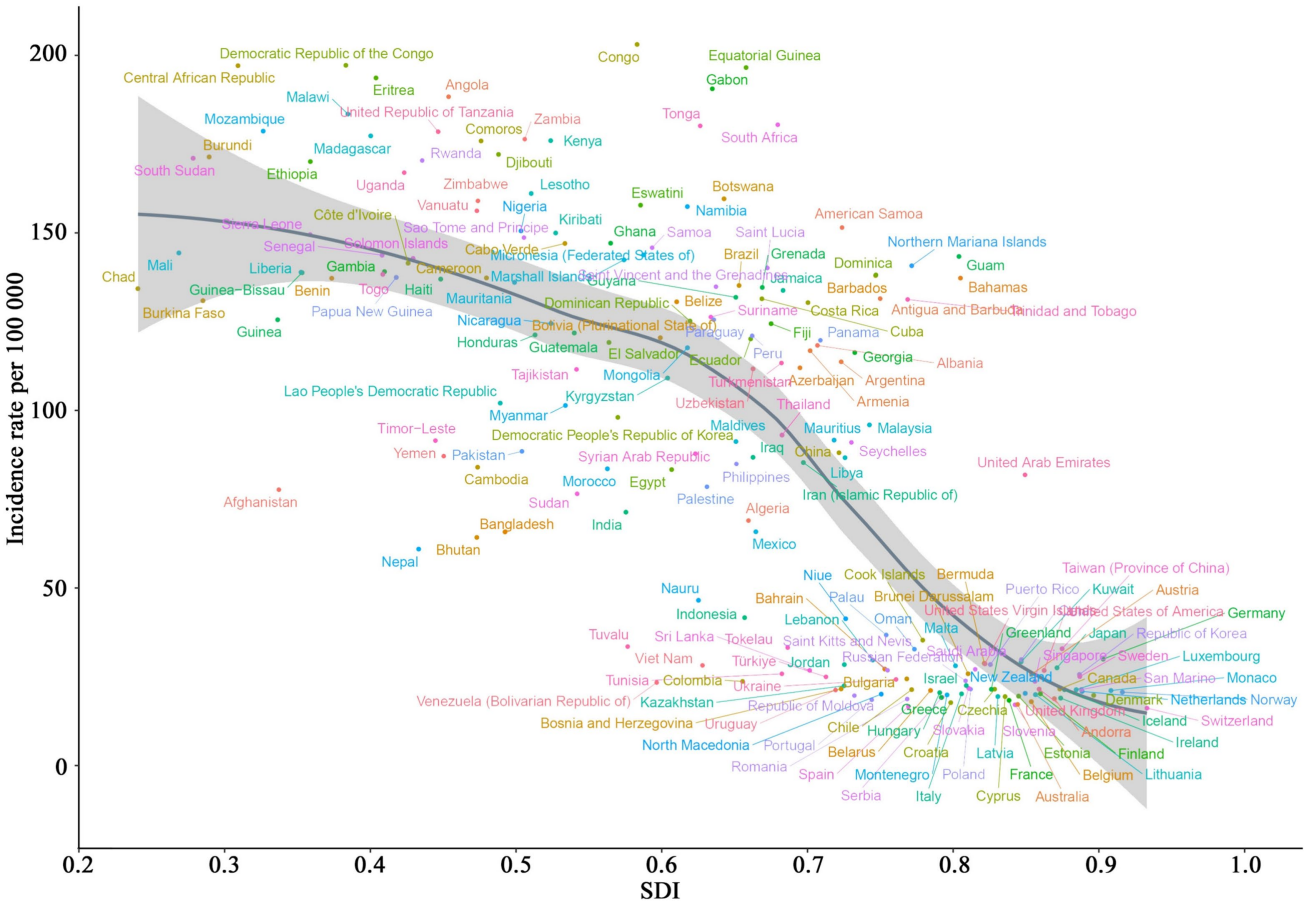
The association between incidence rates and SDI across 204 countries (1990–2021).

**Figure 7:**
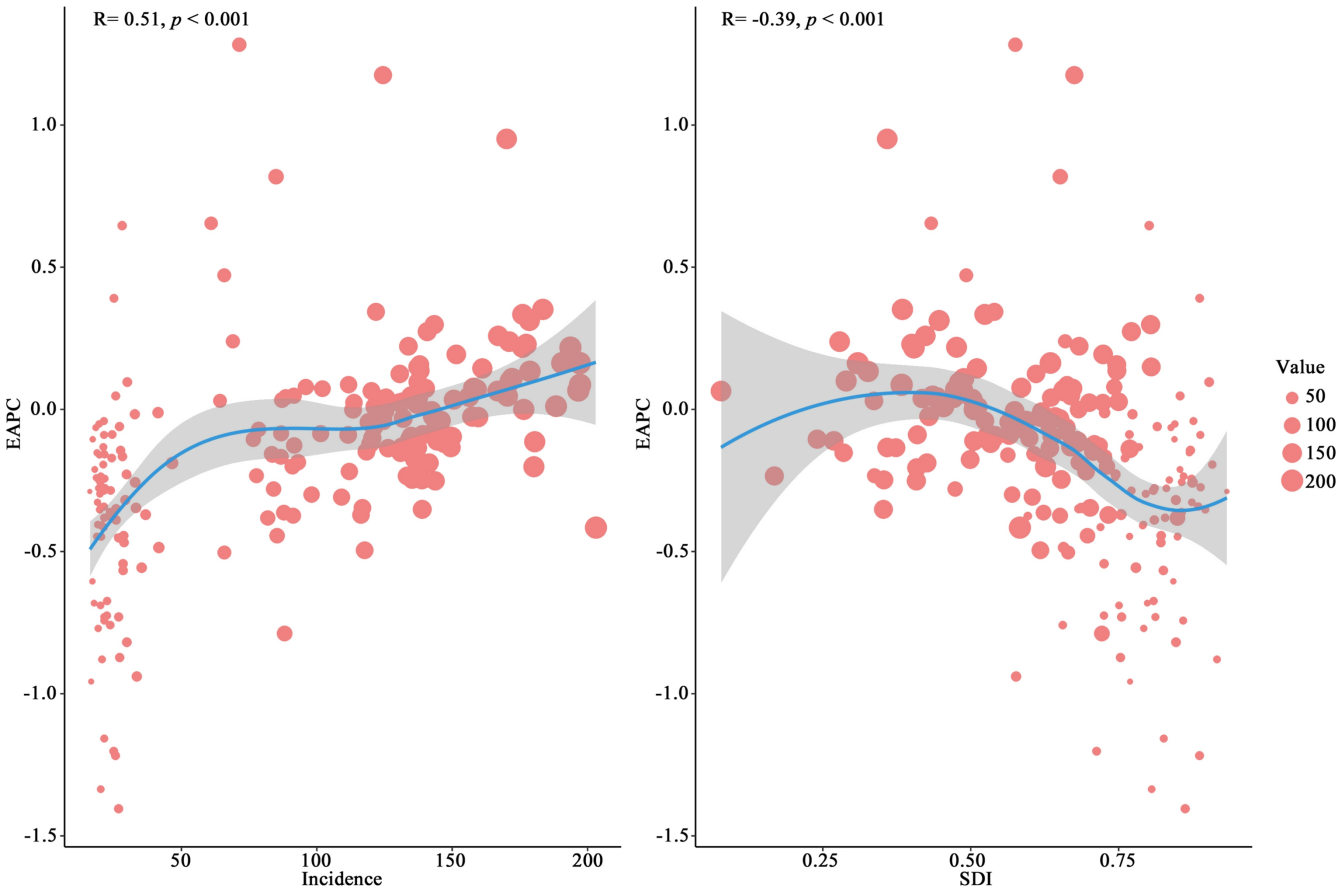
Association analysis of CCVD incidence rate, SDI, and EAPC (1990–2021).

An overall negative relationship between incidence rates and SDI is illustrated in Figure 5 across the analyzed regions. Low-SDI regions, such as Central Sub-Saharan Africa and Eastern Sub-Saharan Africa, exhibited the highest incidence rates, exceeding 156.67, while high-SDI regions (e.g., Australasia and Western Europe), showed significantly lower incidence rates, dropping below 23.95. Some regions, such as South Asia, displayed substantial fluctuations, whereas certain middle-SDI regions, such as Central Latin America, maintained relatively stable incidence rates without significant declines. Figure 6 demonstrates the distribution of incidence rates and SDI at the national level. Low-SDI countries (e.g., Democratic Republic of the Congo and the Central African Republic), clustered in the higher incidence rate range, whereas high-SDI countries, such as Switzerland and Australia, were concentrated in the lower incidence rate range. Notably, even among countries with similar SDI levels, there were considerable differences in incidence rates. Figure 7 explores the relationship among incidence rate, SDI, and EAPC. Incidence rate and EAPC are positively correlated (*r* = 0.51, *p* < 0.001), with countries exhibiting incidence rates below 50 generally having EAPC values concentrated between −0.5 and 0. SDI and EAPC are negatively correlated (*r* = −0.39, *p* < 0.001), with the relationship being particularly pronounced in countries with SDI values ranging from 0.40 to 0.70. The lowest EAPC was observed at an SDI level of 0.90. SDI plays a crucial role in shaping disease burden patterns, as countries with low SDI levels persistently face rising CCVD burdens.

### 3.6 Attribution of CCVD mortality to major risk factors

**Figure 8:**
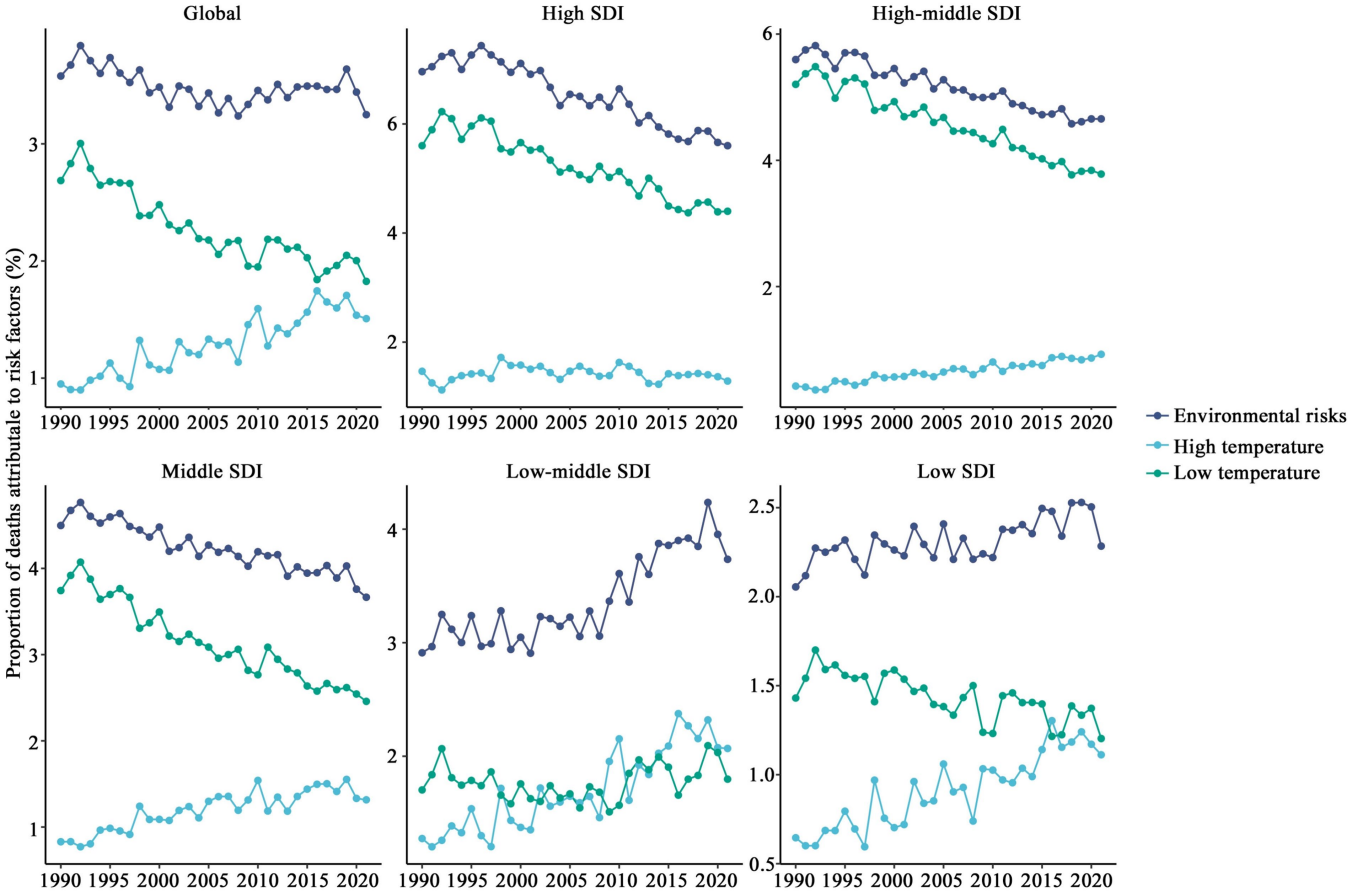
Changes in CCVD-related mortality attributed to major risk factors.

**Figure 9:**
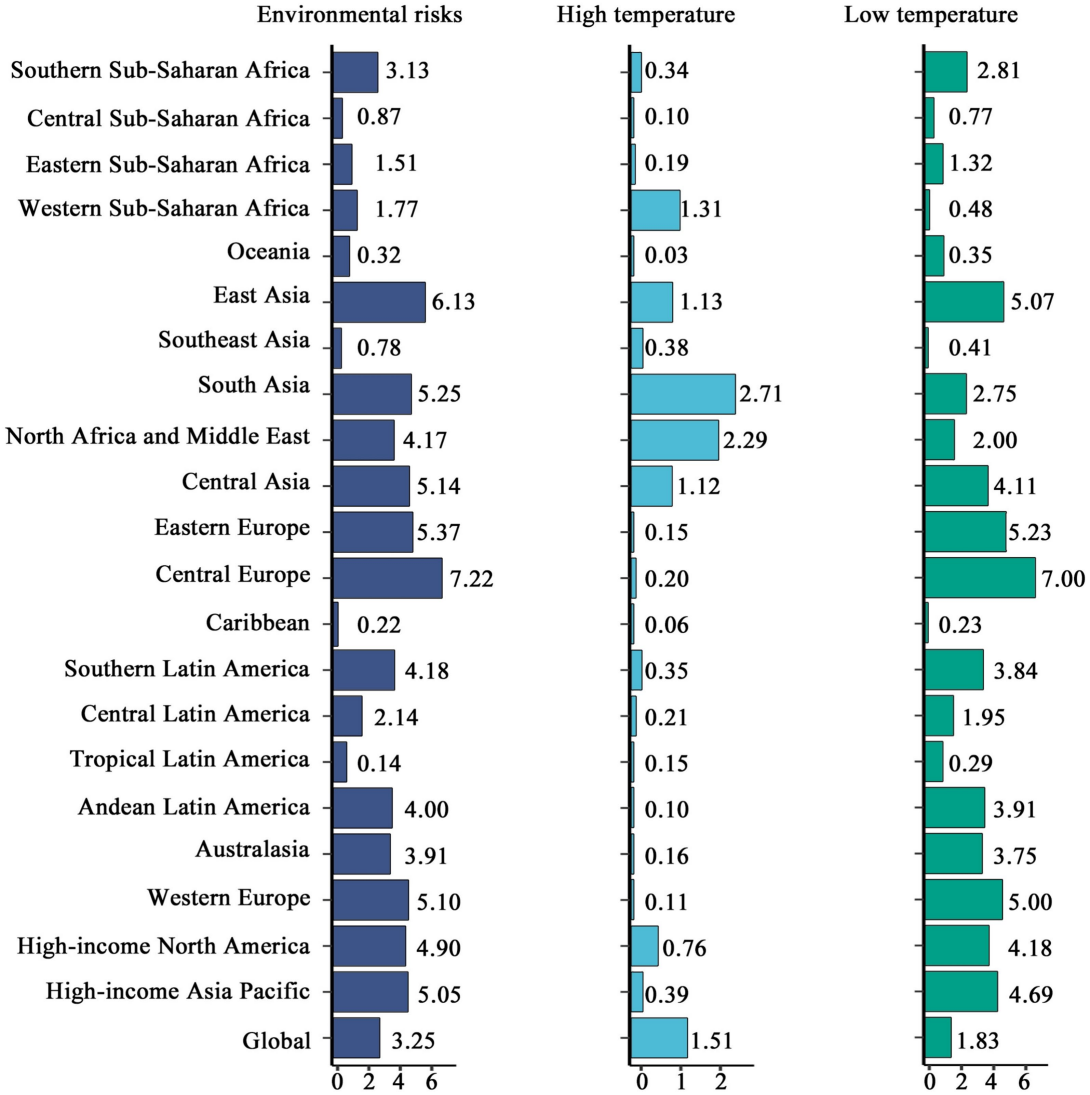
Comparative analysis of CCVD mortality attributed to major risk factors across regions (2021).

Environmental risks have a significant impact on the mortality percentage of CVD globally (see Figure 8). The contribution of environmental risks to mortality has remained relatively stable at around 3.20%, with minimal fluctuations over time. The contribution of low temperatures to mortality has consistently exceeded that of high temperatures, although the weight of high temperatures has gradually increased over time, while the contribution of low temperatures has decreased. Environmental risk contributions have been rising in low and low-middle SDI regions, with the impacts of low and high temperatures gradually converging. As shown in Figure 9, in tropical and impoverished regions, environmental risks are the primary causes of mortality. For instance, Southern Sub-Saharan Africa (3.18%), South Asia (5.25%), and East Asia (6.18%) exhibit a notable impact from high temperatures, though their overall proportion remains lower than in regions dominated by low temperatures. In temperate and cold regions, low temperatures are the predominant risk factor, such as in Eastern Europe (5.23%), Central Europe (7.04%), and East Asia (5.07%). Overall, while there has been a significant global decline in environmental risks, the proportion of risk remains higher in impoverished regions, like Southern Sub-Saharan Africa and South Asia.

## 4 Discussion

### 4.1 Trends and regional disparities in CCVD incidence

This study found a steady global increase in CCVD incidence from 1990 to 2021, accompanied by a moderate decline in mortality, with the most significant disparities observed across SDI levels, age groups, and gender. Worldwide, the incidence of CCVD has steadily risen by about 25.27%, with marked disparities across regions stratified by SDI levels. Regions with high and high-middle SDI levels exhibited declining trends in both CCVD incidence and EAPC, at −0.77 and −0.83, respectively. This decline reflects significant achievements in improving child health through advanced healthcare systems and effective preventive interventions. In contrast, CCVD incidence continued to rise steadily in low and low-middle SDI regions, with EAPCs reaching 0.66 and 0.45, respectively, highlighting critical gaps in healthcare provision, environmental control, and safety infrastructure (12, 13). The study also revealed that CCVD incidence rates were higher among female children compared to males, the disparity between genders grew over the study period and was especially evident among individuals aged 10 to 14. Such findings may result from pubertal physiological transformations and the increased likelihood of associated health complications in the female population. Meanwhile, a steady reduction in CCVD incidence among children younger than 1 year suggests the success of international health interventions aimed at early childhood. However, incidence rates among children aged 5–14 have been on the rise since 1996, with the 10–14 age group emerging as the most affected. This pattern may be linked to the emergence of metabolic disorders, poor health behaviors, and dietary imbalances during early developmental stages. Regional analysis identified Southern Sub-Saharan Africa and South Asia as the areas with the heaviest CCVD burdens. For instance, the Republic of Congo recorded the greatest incidence rate (203.09 per 100,000), while India and Fiji Showed the sharpest growth in incidence (EAPC 1.29 and 1.76, respectively). These high rates are closely linked to shortages in medical resources, unsafe environmental sanitation, and increased exposure to environmental risks (12, 13). In contrast, high-income countries such as Spain and Switzerland reported the lowest incidence rates (16.65 and 16.17 per 100,000, respectively), reflecting advanced healthcare systems and robust health management capacities. However, it is noteworthy that some high-income countries, such as Sweden, despite having a relatively low incidence rate (25.06 per 100,000), displayed an upward trend in EAPC (0.39), warranting further attention to potential underlying risks. The findings further highlight significant global health inequalities in CVDC. In low-income countries, CVDC incidence continues to rise, reflecting notable deficiencies in health resource allocation and management (14–16). Additionally, global challenges such as climate change and environmental pollution exacerbate the CVDC burden in these regions. Addressing these inequalities remains a critical goal for future efforts, emphasizing the necessity of personalized health intervention strategies.

For countries with higher SDI levels, in addition to continuously improving public health management systems, attention should be focused on the health risks of children aged 10–14 years, with particular emphasis on health interventions for adolescent girls. For countries with lower SDI levels, it is imperative to enhance basic healthcare infrastructure and health management strategies. This includes strengthening environmental risk prevention, improving living conditions, reducing children’s exposure to unsafe environments, promoting health education, and addressing CVD risks arising from metabolic disorders. Global collaboration is essential, with countries providing mutual support through appropriate technical and financial assistance to high-risk regions and populations.

### 4.2 Global trends and regional disparities in CCVD mortality

Significant disparities in mortality rates across global regions were reaffirmed by the study. CCVD mortality rates increased slightly worldwide between 1990 and 2021, with the most notable growth occurring in low-SDI countries. This trend is primarily associated with non-optimal temperatures, particularly cold exposure. These areas often lack adequate basic healthcare infrastructure (17, 18) and sufficient heating facilities, making children more vulnerable to cardiovascular health risks posed by environmental and temperature factors. From a biological perspective, exposure to low temperatures can cause vasoconstriction and increased peripheral resistance, leading to elevated blood pressure and increased cardiac workload. Low temperatures also activate the stress-related sympathetic nervous system, affecting heart rate variability and blood viscosity, and in severe cases, may induce arrhythmias, thereby increasing the risk of CVD. Exposure to cold poses heightened risks for children due to their increased physiological susceptibility due to their underdeveloped thermoregulatory capacity, faster metabolism, and immature peripheral circulatory system. This vulnerability is further exacerbated in regions with inadequate cold protection measures and limited access to medical interventions, where the adverse effects of low temperature on CCVD are more pronounced. Conversely, a downward trend in CCVD mortality was observed in areas with high SDI. This can be attributed to advanced preventive treatments for CVD and stronger adaptability to cold temperatures in these regions (14). Globally, low temperatures remain the predominant driver of CCVD mortality associated with non-optimal temperatures, particularly in cold regions like Eastern Europe, Central Europe, and East Asia. Compared to high temperatures, low temperatures place greater stress on the cardiovascular system, potentially causing elevated blood pressure, vascular constriction, and other adverse reactions. However, in tropical and low-SDI regions, the impact of high temperatures is steadily increasing, particularly in South Asia and Southern Sub-Saharan Africa, a trend closely tied to intensifying climate change and rising heat exposure (19, 20). Regional assessments indicate that low-income regions, including Southern Sub-Saharan Africa and South Asia, carry the greatest CCVD burden as a result of high environmental risk exposure, inadequate healthcare infrastructure, and insufficient health system management (21). In temperate regions like Eastern and Central Europe, despite relatively higher economic levels, cold climates make low-temperature risks a significant factor in CCVD mortality. While CCVD mortality remains low in high-income nations like Western Europe and Australia, rising heat-related risks are becoming more prominent and deserve closer scrutiny (22). The results emphasize the necessity of implementing tailored CCVD prevention and control measures. In nations with low SDI, such interventions are particularly urgent due to systemic healthcare limitations and elevated environmental risks, ensuring environmental safety and improving child-oriented healthcare management systems are critical. In colder regions, enhancing heating infrastructure is essential to mitigate the risks posed by low temperatures to CCVD. Conversely, in hotter regions, efforts should focus on strengthening heat warning systems, outdoor shading, and cooling infrastructure (23). These measures are vital to promoting equitable development in global child health.

### 4.3 Strengths and limitations

The WHO’s Global Action Plan for the Prevention and Control of NCDs 2013–2030 and UNICEF’s Strategic Plan 2022–2025 both emphasize the importance of early childhood interventions in preventing CVD and other non-communicable diseases (NCDs), and call for stronger integration between child health and environmental adaptation policies (24). In recent years, many national governments have incorporated childhood cardiovascular health risks into their national NCD prevention strategies. For example, the United Kingdom’s Childhood Obesity: A Plan for Action, the United States CDC’s National Childhood Obesity Prevention Program, and China’s Healthy Development of Children and Adolescents (2019–2030) all emphasize the critical need for early screening and timely management of childhood hypertension, obesity, and metabolic dysfunctions (25–27). In response to extreme climate risks, Germany’s Klimawandel und Gesundheit has integrated the health impacts of temperature exposure on children into the national public health emergency framework. France’s Heatwave Plan designates children as a priority population in heatwave intervention strategies, and countries such as Japan have established comprehensive climate adaptation policies specifically for children, addressing both cold and heat stress. The findings of this study provide global epidemiological evidence to support the refinement and implementation of such policy frameworks (28, 29). They also call for more countries to incorporate CCVD risks into their national NCD prevention and control strategies—such as introducing child-specific risk levels in extreme weather alert systems, promoting international collaboration, institutionalizing community-based cardiovascular health screening for children, and integrating CVD risk indicators into routine monitoring systems. Furthermore, there is a need to develop national and global CCVD health data management platforms to enable long-term tracking and predictive modeling of childhood cardiovascular risks (30, 31).

Data from the GBD study—spanning 30 years and covering 204 countries and territories—was employed in this analysis, providing a robust foundation for systematic analyses at global, regional, and SDI levels. Children aged 0 to 14 years account for approximately 25.2% of the global population; thus, developing targeted prevention and control strategies for CVD in this age group is a critical step toward reducing health disparities and achieving health equity. This study is the first to systematically present the global distribution and temporal trends of CCVD burden, offering in-depth analyses of disparities by gender, age group, and country or region, thereby contributing to the advancement of comprehensive and equitable child health outcomes.

However, several limitations should be noted. (1) GBD estimate accuracy is largely dependent on the completeness and reliability of national health data, which vary across regions. In particular, CCVD data in low-SDI countries are often incomplete or of lower quality, primarily due to insufficient screening coverage and underdeveloped data registration systems. Although various modeling techniques are employed to minimize bias and uncertainty, such limitations cannot be entirely eliminated. (2) The attribution of environmental risk factors is based on model-derived estimates, which may not fully capture the contextual variability in social environments or individual-level exposures across countries. (3) This study is ecological in nature and thus cannot infer causal relationships at the individual level. (4) There is currently no internationally standardized classification system specifically for CCVD subtypes. Future research should incorporate more detailed and disaggregated data to support the development of such a classification system and to provide a stronger evidence base for relevant prevention and control strategies.

## 5 Conclusion

This study provided a comprehensive analysis of global trends in childhood cardiovascular disease (CCVD) incidence and mortality between 1990 and 2021, uncovering significant disparities in disease burden across regions, socio-demographic index (SDI) levels, genders, and age groups.

First, the global incidence of CCVD exhibited a gradual increase, particularly in low- and low-middle SDI regions, where both incidence rates and estimated annual percentage changes (EAPC) continued to rise. Key drivers included environmental risks, exposure to non-optimal temperatures (high and low), and insufficient healthcare resources. High and high-middle SDI countries experienced substantial decreases in incidence rates, primarily resulting from strong healthcare frameworks and the implementation of preventive health strategies. Analysis of gender and age differences indicated consistently higher incidence rates among females, with the gender gap expanding over time, most notably in the 10–14-year age range.

Second, regional and risk factor-related differences in mortality were equally pronounced. Marked reductions in CCVD mortality were observed in high-SDI regions, largely driven by progress in medical technologies and enhanced environmental management. However, mortality rates in low-SDI regions remained persistently high, driven primarily by environmental risks and low-temperature exposure. Meanwhile, in tropical regions, the impact of high temperatures is gradually increasing. Age group analysis showed a significant reduction in mortality among children under 1 year of age, but CCVD-related mortality increased within the 5–14 age bracket, with the 10–14 subgroup experiencing the greatest impact.

Overall, the imbalance in the global CCVD burden across SDI levels and risk factors reflects disparities in public health resource allocation and disease prevention capacities. Prioritizing interventions in low- and lower-middle-income regions is reinforced by the study’s findings, including improving healthcare infrastructure, enhancing environmental risk management, and increasing children’s health education. For high-income countries, attention should be directed toward the potential growth in CVD burden among adolescents, with efforts to optimize health monitoring and preventive strategies.

CCVD control is critically important within the global strategy for non-communicable disease (NCD) prevention. The application of targeted health policies, along with reinforced international partnerships to alleviate the global CCVD burden, plays a vital role in mitigating non-communicable diseases and enhancing child well-being, thereby aligning with the United Nations Sustainable Development Goals.
